# Cutting Edge PBPK Models and Analyses: Providing the Basis for Future Modeling Efforts and Bridges to Emerging Toxicology Paradigms

**DOI:** 10.1155/2012/852384

**Published:** 2012-07-30

**Authors:** Jane C. Caldwell, Marina V. Evans, Kannan Krishnan

**Affiliations:** ^1^National Center for Environmental Assessment Office of Research and Development, U.S. Environmental Protection Agency, 1200 Pennsylvania Avenue, Washington, DC 20460, USA; ^2^National Health and Environmental Effects Research Laboratory, Office of Research and Development, U.S. Environmental Protection Agency, Research Triangle Park, NC 27711, USA; ^3^Department of Occupational and Environmental Health, Université de Montréal, Montreal, QC, Canada H3C 3J7

## Abstract

Physiologically based Pharmacokinetic (PBPK) models are used for predictions of internal or target dose from environmental and pharmacologic chemical exposures. Their use in human risk assessment is dependent on the nature of databases (animal or human) used to develop and test them, and includes extrapolations across species, experimental paradigms, and determination of variability of response within human populations. Integration of state-of-the science PBPK modeling with emerging computational toxicology models is critical for extrapolation between *in vitro* exposures, *in vivo* physiologic exposure, whole organism responses, and long-term health outcomes. This special issue contains papers that can provide the basis for future modeling efforts and provide bridges to emerging toxicology paradigms. In this overview paper, we present an overview of the field and introduction for these papers that includes discussions of model development, best practices, risk-assessment applications of PBPK models, and limitations and bridges of modeling approaches for future applications. Specifically, issues addressed include: (a) increased understanding of human variability of pharmacokinetics and pharmacodynamics in the population, (b) exploration of mode of action hypotheses (MOA), (c) application of biological modeling in the risk assessment of individual chemicals and chemical mixtures, and (d) identification and discussion of uncertainties in the modeling process.

## 1. Introduction: PBPK Modeling

After exposure of environmental pollutants or pharmaceuticals to humans, experimental animals and, where relevant, cellular systems, potential adverse effects are dependent on an agent's toxicokinetic information (absorption, distribution, metabolism, and elimination (ADME). Physiologically based pharmacokinetic (PBPK) models are a class of biological models that utilize this information to translate external exposures into an internal (target) dose in the body. PBPK models are not only often used to predict toxicologically relevant internal doses, but also to account for any nonlinearities between internal and external applied dose or exposure. Traditionally, these models have been used for performing extrapolations between different routes of exposure and between different species; they use species specific anatomical, physiological, chemical-specific, and biochemical parameters [1, 2]. However, parameters may vary between species and chemicals of interest (e.g., solubility and metabolic transformation rates within tissues of the body) and the accuracy of any PBPK model dependent on the accuracy of its parameter information [3]. Not only are metabolic parameters species-specific, but in many cases considerable parameter variability exists between individuals within populations. The impact of parameter variability on predictions of toxicologically relevant doses can be estimated through PBPK model configuration changes. Although thought to be greater in humans than in the test animals often used to test toxicity, the impact of variability within species can be estimated by sensitivity and uncertainty analyses of PBPK models. 

PBPK models describe the mass balance of materials within and between various tissue compartments. In terms of model structure, if the transfer of substances across the capillary and cellular membrane is very rapid compared to the perfusion rate of the tissue, then the three compartments can be collapsed into one homogeneous compartment depicting the whole organ. In these cases, the model is called a “flow-limited” model, and the ratio of concentration between the blood and the organ (partition coefficient) is governed by the solubilities in each tissue and metabolic processes within the organ [3]. Therefore, the disposition of a chemical throughout the body is governed by partitioning between organs and blood, partitioning between blood and air, blood flows to organs, ventilation rates, absorption rates, metabolic rates, and elimination rates [3].

Toxicological applications for PBPK modeling have been increasing over the last 30 years. The many applications and role of this science include determining: (a) environmental exposure from sampling of parent and/or metabolite(s) in tissue and biological fluids, (b) target organ or system concentrations of parent and/or metabolite(s) from exposure, (c) the appropriate dose metric from mode of action (MOA) information, and (d) the use of PBPK modeling to test hypothesis regarding the effects of metabolic variation. When human data (e.g., pharmacokinetic and exposure) are available, PBPK models can be used directly to transform external exposure estimates into internal doses. When experimental animal data are only available, extrapolation uncertainty can be reduced by the use of an appropriate PBPK model. However, this reduction in one type of uncertainty may be outweighed by the introduction of other types of uncertainty associated with the structure and assumptions inherent in the modeling. Such uncertainty includes the limitations of describing the scope and nature of variability of pharmacokinetics and pharmacodynamics of an agent within the human population. Human variability could be due to differences among individual adults, across genders, or between life stages. In support of human health assessments, a key expectation of future PBPK modeling efforts increased ability to accommodate increases in knowledge of population variability in a number of areas, such as target organ structure, types and numbers of cells at risk to toxicant exposure, age- and gender-specific differences, and human activity patterns. In this way, mechanistic and mode of action (MOA) explanations can be linked to quantified measures of exposure. 

**Figure 1:**
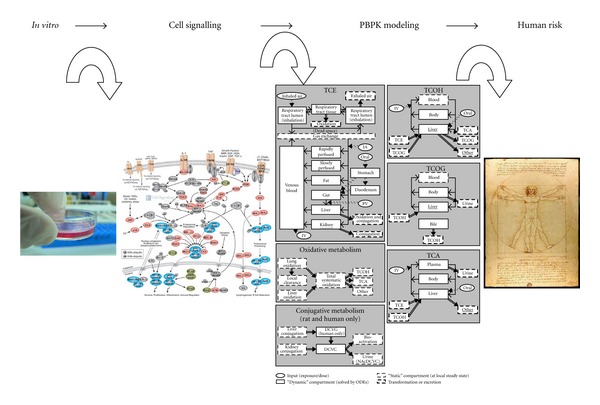
This figure represents, in simplistic fashion, major extrapolations between data that has been derived from *in vitro* isolated cell systems to interpretations of that data for cell-signaling pathways, the extrapolation of cell signaling results in regard to PBPK models that assign appropriate dose metrics and target organ concentrations, and finally the prediction of human risk from resulting models for a particular xenobiotic exposure.

The cover of this special issue (i.e., shown below in Figure 1) illustrates key extrapolations made between the cellular *in vitro* responses from single cells to risk of health effects in humans. These steps include (1) interpretation of *in vitro* cellular signals within a complex network of signaling pathways at the cellular level (i.e., the pathway shown is for NF*κ*B which is relatively complex and context dependent on its action [4]), (2) construction of a validated PBPK model (i.e., the recently published PBPK model for trichloroethylene which represents a complex and state of the art effort), and (3) extrapolation of internal dose and response of a target organ or system in DaVinci's “ideal man.” While such a prediction to the ideal or “average” individual is often the reported result, such predictions are not representative of sensitive subpopulations that are epigenetically and genetically diverse, and more liable to express an endpoint of concern resulting from concurrent exposures, gender, age, and stage of development. The goals of this editorial overview are threefold: (1) to highlight the principles of best practices in PBPK models intended for risk assessment application, (2) to provide a perspective on the articles in the special issue, and (3) to present our viewpoints of what represents the cutting edge of science in this field and what specific research needs and approaches may be useful in making progress towards future applications. 

## 2. Best Practices 

Computational models of toxicological processes are developed based on hypothetical or proven interrelationships among critical processes and parameters. However, before they can be used confidently in risk assessments or other applications, it is essential that the model structure, parameters, and performance are evaluated systematically. Even though the terms—model validation and model evaluation—are used interchangeably or preferentially, they refer to whether a model with a given set of input parameters can reasonably reproduce the system behavior for a defined set of conditions, and whether the key determinants of the system behavior have been adequately captured by the model [5]. Often, the developers of the model and those who apply the model have different views and expectations in regard to model validation. 

An important aspect in assessing the performance of a PBPK model is how well it fits several data sets. It is important to be able to understand and characterize the strengths, limitations, and relevance of the data to the endpoint(s) of concern, and to choose measured endpoints for comparison with predicted results. The accuracy and precision of the methods used in each study used to provide that data and the apparent reproducibility of results must be examined so that such judgments can be made. Variation of results reported from differing studies may reflect human variability of response, the effects of differing exposure protocols, or differences in the accuracy or precision of measured values in each study [3]. 

Even though there has been extensive focus on a model's ability to provide predictions that match closely with some limited data, the more relevant goal should be to characterize the level of confidence in the model's fit for a specific purpose or end-use. Accordingly, there is increasing emphasis on evaluating a model rather than validating a model (e.g., [6]). If a model has been developed for a specific purpose (e.g., a particular risk assessment application), then relevant aspects of the model should be evaluated in context. In this regard, the International Program on Chemical Safety (IPCS) of the World Health Organization (WHO) has recently published guidelines for characterization and purpose-specific evaluation of PBPK models [7]. A detailed description of the PBPK model evaluation process for use in risk assessment is also described in a recent publication [8]. Accordingly, if the model is to be used for conducting interspecies extrapolation for the oral route based on a particular dose metric (e.g., AUC), then the evaluation should focus on the ability of the model to provide the relevant simulations, and not on generically “validating” the model for all possible applications. Thus, the level of confidence in the use of a PBPK model for a defined and specific purpose in a risk assessment (e.g., prediction of a dose metric for conducting rat to human extrapolation) can be established on the basis of the following [7]: In this context, the following key aspects/questions are evaluated [7].

- Do the model structure and parameters have a reasonable biological basis?
- How well does the PBPK model reproduce the chemical-specific pharmacokinetic data under various experimental or exposure conditions?
- How reliable is the PBPK model with regard to its predictions of dose metrics relevant to risk assessment? In this regard, it is important to evaluate the level of sensitivity of the predictions to the model parameters and the level of uncertainty of the parameter values. Emphasis is placed on sensitivity and uncertainty sensitivity analyses so as to identify the following:
  - the model parameters that most strongly influence the dose metrics associated with human risk assessment conditions (e.g. exposure pathways, relevant exposure conditions such as acute or chronic), and
  - the model parameters that have the most influence on the dose metrics associated with the study or studies from which the critical end-points are derived (i.e. toxicity, epidemiological, and clinical studies).

Documentation of model development is not only essential for evaluation of the key aspects of the model that appear to function appropriately, but also for those that did not during the development process. The following general principles constitute the current state of best practice in PBPK modeling [7–10]. (1) The model should be capable of simulating all potentially useful dose metrics for the exposure routes, lifestages, and doses in the species of relevance to an assessment; (2) the structure of the PBPK model should contain the target organ (or a surrogate tissue) as well as compartments representing tissues of unique physiological and biochemical relevance to the pharmacokinetics of the chemical; (3) the equations chosen to describe ADME should be scientifically supported; (4) the tissue volumes, flow rates, ventilation: perfusion ratios used in the model should be within physiological limits, the sum total of the tissue volumes should not exceed the body weight, and the sum total of tissue blood flow rates should equal cardiac output; (5) partition coefficients for the model should be obtained using *in vitro* methods, *in vivo* data obtained at steady-state, or theoretical algorithms within their boundary of valid application; (6) biochemical parameters for the model should be estimated from *in vivo* data or on basis of adequate scaling of *in vitro* data; (7) solutions to the differential equations in a PBPK model need not to be evaluated if a highly reputable commercial or open source simulation software has been used although an appropriate algorithm should have been selected; (8) the appropriateness of the integration algorithm and integration intervals should be justified, particularly when a new software tool or a custom-made program is used for modeling; (9) evaluation of the model structure and parameters should be conducted to ensure that the model adequately predicts the pharmacokinetic behavior (i.e., bumps and valleys in the concentration versus time plot) of the chemical and that the parameters (point estimates, range of values, or distributions) consistently describe available data; (10) sensitivity, uncertainty, and variability analyses should be conducted using acceptable statistical methods.

## 3. State-of-the Art Application of PBPK Modeling in Risk Assessments

While the toxicological profiles and IRIS values produced by EPA are not complete risk assessments, they provide information on a chemical's potential for causing adverse health effects along with information about the relationship between the dose of the substance and the biological response. When this information is combined with information about exposure, these values are used internationally to characterize the public health risks of chemical substances [11]. PBPK models have been developed for several high profile chemicals that are the subject of EPA IRIS assessments. Recent state-of-the-art analyses have used PBPK models and human and rodent data sets for such applications to high impact and complex risk assessments. They form a bridge for future development of PBPK model applications to even more complex data sets. 

Dichloromethane (methylene chloride or DCM) has been modeled to have two pathways of metabolism (i.e., oxidative and GSH conjugation pathway) with the GSH pathway assumed to be responsible for its carcinogenicity [12, 13]. Alternatively for one of the most important P450 isozymes (CYP2E1) in the toxicology of environmental exposures, new PBPK models and a reexamination of *in vitro* and *in vivo* data show that DCM metabolism can primarily occur through this enzyme via the oxidative pathway, but with two sites for DCM metabolism [14, 15]. Trichloroethylene (TCE) is a widespread environmental contaminant with complex metabolism that also involves the same two pathways [16]. Both pathways of metabolism are a crucial component of its toxicity, particularly in liver (via the oxidative pathway) and kidney (via the GSH pathway). A state-of-the-art PBPK model for TCE with detailed lung compartments, extensive rodent and human datasets, and Bayesian analyses have been used to (1) characterize uncertainty and variability in these metabolic pathways, and (2) to describe the complex mixture of internal exposures of metabolites linked to TCE's MOA in several target organs that includes predictions of GSH conjugation and bioactivation in the kidney [16–18]. 

While metabolism is usually limited by the blood flow into the liver of the parent compound (i.e., flow-limited), in the case of methyl tertiary butyl ether (MTBE) metabolism is limited by the ability of the enzyme to metabolize the parent compound (i.e., enzyme-limited) [3]. In this case, human microsomal data in combination with an updated PBPK model may be used to (1) predict human variability for dose metrics potentially associated with human response, and (2) to test hypothetical scenarios representative of the range of human metabolism of MTBE [3]. This example illustrates the use of microsomal metabolism differences in conjunction with PBPK models as a more robust tool than microsomal metabolism differences alone to predict differences in risk.

Evans and Caldwell [14] brought forth that the use of two binding sites for CYP2E1 has the potential not only to account for the broad range of chemicals that it is able to metabolize, but also inferences regarding PBPK modeling and the use of chamber data to discern pharmacokinetics and MOA. The two-binding-site work by Evans and Caldwell [14] was commented on a letter to the editor authored by Anders et al., [19] followed by a response by Evans and Caldwell [15]. In this Special issue Cuello et al. use a similar approach to analyze inhalation chamber data, metabolism data (CYP2E1 and GSH conjugation), and a PBPK model to examine the plausibility of whether two sites on the same enzyme or two separate enzymes can account for *in vivo* metabolic clearance profiles of bromochloromethane, a brominated disinfection byproduct. For both metabolic hypotheses, sensitivity analyses of *in vivo* experimental data are used to evaluate model parameter impacts on predicted outcomes, and to guide the design of future experiments needed to fully address the metabolic mechanisms involved for this specific chemical. 

As the available database and PBPK models for a particular chemical can be complex, another layer of complexity can be added from the existence of internal and external metabolite exposure (e.g., TCE and its metabolites) or coexposures to similar compounds. In this special issue, Sasso et al. present a lipid-based PBPK model for the analysis of a mixture of six polychlorinated biphenyls (PCBs) in rats. Population Bayesian analysis was applied that incorporated an internal exposure-response model linking enzyme induction and metabolic rate. The PBPK model was specialized to simulate concentrations of highly lipophilic compounds in tissue lipids without the need for partition coefficients. In addition, a hierarchical treatment of population metabolic parameters and a CYP450 induction model were incorporated, and Markov-Chain Monte Carlo simulation applied. For all dose levels and dose profiles, the model predicted PCB concentrations in multiple tissues. This specific computational technique provides an alternate approach for analysis of compounds for which partition coefficients are not experimentally available (e.g., as is the case for most nonvolatile compounds). 

After exposure, a chemical's MOA and background metabolite exposure affects the selection of an appropriate dose metric for modeling internal concentrations in target organs or systems. Along with differences in pharmacokinetic parameters, there is variability in response to a particular dose metric concentration within the human population due to a number of factors that include developmental status and age at exposure (i.e. children versus adults). The differences in pharmacokinetic parameters and development of PBPK models between potential sensitive human subpopulations and/or life stages are the subject of several papers in this Special issue.

In 2008, Health Canada concluded that (1) while recent developments in PBPK modeling of manganese were important and informative, the science was not appropriate at that point in time for establishing a health-based reference concentration for inhaled manganese, and (2) a validated, peer-reviewed human inhalation PBPK model for manganese parameterized for the various subgroups of concern was needed [20]. These subgroups include neonates, iron-deficient individuals, and others with certain medical conditions such as cholestatic liver disease [21, 22]. In this special issue, Dorman et al. present a review of the topic as it relates to generation of pharmacokinetic information on the inorganic manganese combustion products of the organometallic fuel additive methylcyclopentadienyl manganese tricarbonyl (MMT) in compliance with the test rule under the US Clean Air Act. The Alternative Tier 2 testing program for MMT is described with emphasis on the development of pharmacokinetic data and generation of PBPK models for manganese. In the companion paper, Taylor et al. review (1) the development of PBPK models for experimental animals and human at various stages of development, (2) relevant risk assessment applications of the models, and (3) model predictions of manganese tissue concentrations for individuals with altered physiology due to life stage or condition including age (e.g., fetal, neonatal), pregnancy status, liver disease, or chronic inhalation exposure to manganese. Applications of such model predictions include the development of uncertainty factors for use in risk assessments that take into account these populations.

The impact of variability in human whole and subpopulations on uncertainty factors was also examined by Valcke et al. in this special issue; human kinetic adjustment factors (HKAF) were discussed for inhaled volatile organic chemicals (VOCs). Population distributions (i.e., for adults, elderly, children, neonates and pregnant women) of blood concentrations and rates of metabolism were generated by Monte Carlo simulations to a steady-state algorithm for Benzene and 1,4-dioxane (1,4-D) exposure. For these specific blood-flow-limited volatiles, blood concentration-based HKAFs were the most affected in distinct subpopulations (i.e, blood concentration having a greater effect than rates of metabolism).

For one of the three case studies presented for PBPK model applications in risk assessment, the specific effects of age are also reported by Mielke and Gundert-Remy in this special issue. In the first case study, lower enzyme expression levels in newborn infants are used to estimate bisphenol A (BPA) blood levels near the TDI for the oral exposure as calculated by the European Food Safety Authority (EFSA). In another case study, adult risk is reported from dermal exposures to BPA. Finally, after dermal exposure to coumarin via cosmetic products, PBPK modeling was used to identify liver peak concentration (the dose metric used for liver toxicity). Dermal and oral exposure pathways were compared. In these cases PBPK modeling was useful to support risk assessments.

When linked with biomarker data, the reconstruction of exposure dose using PBPK modeling has been offered as a valuable tool. However, as noted by McNally et al. in this special issue, due to the lack of exposure and kinetic data, the correlation of biomarker levels with exposure concentrations leads to difficulty in utilizing biomonitoring data for biological guidance values. McNally et al., use exposure reconstruction (i.e., reverse dosimetry), PBPK modeling, global sensitivity analysis, Bayesian inference, and Markov chain Monte Carlo simulation to obtain a population estimate of inhalation exposure to *m*-xylene. The importance of model structure and dimensionality is also examined with respect to its ability to reconstruct exposure. 

 The example of chlorpyrifos illustrates the need to take into account differences in susceptibility and the need to understand background exposures of its metabolites in children to reconstruct exposure through PBPK modeling. The PBPK model of Lu et al. [23] had limited success in predicting exposure from urine measurements due to the combination of different sources of exposure. However, the PBPK model for chlorpyrifos was a valuable tool when describing urine in children having ingested specific known amounts of chlorpyrifos. Using urinary biomarker data as the input, Lu et al. report in this special issue the development of a simplified pharmacokinetic model (SPK) for estimation of absorbed doses of chlorpyrifos. Of note, the dose estimates using the SPK model for individual children were significantly higher than those from the conventional PBPK modeling using aggregate environmental measurements of chlorpyrifos as the inputs. 

As demonstrated by the work of Lu et al., simplifications of PBPK models are being developed, but the types of data used to support such efforts are not always available. There is a need for simplified modeling approaches that are still valid for use in risk assessment. While all risk assessments involve uncertainty related to extrapolations from study data to human risk from environmental or pharmaceutical exposures, PBPK models themselves contain two types of uncertainty [3]. Model uncertainty refers to the lack of knowledge needed to determine whether the scientific theory on which a model is based is correct (e.g., alternative choices for model structure, dose metrics, extrapolation approaches, and the appropriateness of surrogate data as in inferences about children from adult data). Parameter uncertainty refers to lack of knowledge about the values of a model's parameters which leads to a distribution of values for each parameter; this uncertainty includes random measurement errors, systematic measurement errors, the use of surrogate data instead of direct measurements, misclassification of exposure status, random sample errors, and the use of an unrepresentative sample. Simplified PBPK model development will need to be specific enough for the types of compounds and scenarios modeled to address both of these types of uncertainty.

For use in site-specific health assessments, Mumtaz et al. (this special issue) report progress on the ATSDR's initiative to develop a PBPK tool box. This tool is designed to contain a series of published models coded in Berkeley Madonna. Ongoing efforts focus on producing a Web linkage to a PBPK database and models that can be accessed for use in assessment activities. In addition, Mumtaz et al. present examples of PBPK model applications that led to (1) derivation of minimal risk levels (MRLs), (2) risk assessment of mixtures, (3) assessment of occupational exposures, (4) site specific assessment, and (5) interpretation of human biomonitoring data.

Finally, in another current application of PBPK models, data from a single patient are used for a clinical case study. In this special issue, the brief communication of Huynh-Delerme et al. is a good example of this type of practical application of PBPK modeling. Potential ethanol exposure was predicted from inhalation or dermal uptake after repeated use of an ethanol-based hand sanitizer. The study investigated whether blood concentrations could be correlated with the incidence of acute pancreatitis in a single patient potentially exposed in the classroom. Thus, this example illustrates another way of applying PBPK models in characterizing the association between health effects and chemical exposures in humans.

## 4. Bridges and Limitations for Future**** Applications

Extrapolation between rodents and humans of the pharmacokinetic behavior of an agent, using only differences in a power of the body weight, inherently assumes that metabolic clearance is scaled consistently across species. However, this assumption does not hold for all dose metrics and enzyme systems [7], and issues arise as to how appropriately scale across species. Extrapolation based on appropriate dose metrics simulated with PBPK models for the test and target species is the preferred approach [7, 24].

Most PBPK models have been developed and tested with experimental animal data and used for extrapolation purposes. Increasingly, this type of data is less available and *in vitro* data or high throughput data from gene expression microarrays are being developed as replacements for animal testing [25, 26]. Although in place for about 10 years, microarray data is no longer the cutting edge with RNA sequencing taking its place for exploration of what types of responses and genetic predispositions lead to cancer and noncancer outcomes. Cell-signaling and gene expression data must in turn be processed through complex informatics approaches to identify pathways and relationships that can further be understood in a physiological context [27, 28]. For relevant dose metrics, PBPK model predictions provide the bridge between concentrations associated with specific cellular responses *in vitro* for a particular paradigm, and the extrapolation to concentrations at the target system or organ in the whole organism. However, all modeling should have phenotypic anchoring in physiology, toxicology, and sound modeling and mathematics to test and understand the models.

The PBPK modeling approach has also been applied to conducting *in vitro* to *in vivo* extrapolations of equivalent doses on the basis of appropriate dose metrics [29–32]. Whole-body PBPK models have been developed for extrapolating the *in vitro* concentration-response curve to *in vivo* dose response [33]. In the context of the next generation toxicity testing initiatives, more recent development in terms of cellular-level PBPK models (e.g., [34]), and biologically based algorithms to predict partitioning into cells, interstitial fluids, and vascular compartments (e.g., [35, 36]) are likely to facilitate more elegant implementation of the *in vitro* to *in vivo* paradigms.

Such biologically based mechanistic algorithms have been developed for the determination of partition coefficients (PCs) that represent the equilibrium ratio of chemical concentration and are, in turn, key input parameters of PBPK models (e.g., blood : air, tissue : blood). The composition of cells, interstitial fluid, and vascular components along with the physiochemical characteristics of chemicals are used in these algorithms to help predict PCs of new chemicals based on QSARs; the algorithms can then be used to help characterize human variability. Initially based on chemical solubility in water and lipid (i.e., from n-octanol : water PCs or vegetable : water PCs (*P*_*o*:*w*_)), first generation efforts focused on prediction of tissue : blood PCs of VOCs, for which macromolecular binding in tissue and blood is negligible [37, 38]. These algorithms were also used to estimate preliminary PCs (e.g., tissue : air, blood : air, tissue : blood) for nonionized and low molecular weight organic chemicals with (1) negligible protein binding (or assumed to be negligible) and (2) one or more CH_3_, CH_2_, CH, C, C=C, H, Cl, Br, F, benzene ring, or H in benzene ring fragments.

On the basis of the solubility and binding processes that are relevant to both ionic and nonionic forms and found in both intracellular and extracellular matrices, the second-generation algorithms not only predicted PCs, but also distribution coefficients (i.e., the sum of ionized and non-ionized forms of chemical in both matrices) [39, 40]. Most recently, Schmitt [35] and Peyret et al. [41] integrated existing algorithms within a single equation for the prediction of distribution coefficients for drugs and environmental chemicals, respectively. Ionization, lipid solubility, water solubility, and tissue binding characteristics (i.e., binding to tissue proteins, acid phospholipids, plasma proteins and hemoglobin) were used in these integrated algorithms to facilitate the prediction of the PCs. When only chemical solubility in lipids and water determine partitioning, all existing algorithms give the same prediction as that of the first-generation algorithm of Poulin and Krishnan [37]; however, when binding to various components is significant then second generation algorithms have been used as reported for the development of PBPK models for pharmaceuticals [42]. However, despite the availability of algorithms and QSARs for predicting partition coefficients of organic chemicals, the availability of metabolism rates for PBPK model development remains a major limiting factor. Very limited research has been conducted for predicting maximal velocity (*V*_max⁡_) and Michaelis affinity constant (*K*_*m*_) for hepatic and extrahepatic metabolism of chemicals (reviewed in [36]). Even though a molecular structure-based QSAR for hepatic clearance has been developed for integration within PBPK models of VOCs, there has not been any attempt to develop global QSARs or quantitative property-property relationships (QPPRs) for wider applicability and integration within PBPK models.

In this special issue, Peyret and Krishnan used QPPR relationship to predict intrinsic clearance (*V*_max⁡_/*K*_*m*_) for volatile organic chemicals. Estimates of intrinsic clearance were then used to parameterize a PBPK model for predicting a blood concentration time course for a series of chemicals. This approach presented in this paper is an initial attempt to develop global QPPRs for estimating intrinsic clearance for incorporation within PBPK models. The approach allows for generation of pharmacokinetic profiles that are consistent with the level of uncertainty in model predictions of intrinsic clearance. It is important to note the limitations of such estimates in order to provide transparency in the risk assessment context, but also to aid in the design of future studies for reduction of uncertainty.

Human liver tissues, hepatocytes, and microsomes continue to represent useful systems to explore individual differences in metabolism of xenobiotics. However, alone these data are not adequate to predict differences in human responses [43]. Under circumstances where rate of metabolism and not blood flow determines *in vivo* intrinsic clearance [3], variability of metabolism extrapolated from human microsomal data can be informative when coupled with an appropriate PBPK model. However, there is uncertainty with procedures extrapolating metabolic values from *in vitro* systems, especially for enzymes that are membrane bound. Recently, a computational approach for the accurate estimation of metabolic clearance for membrane bound compounds (such as P450s) has been developed to address the complexity of these issues [44]. 

Given the desire to reduce the use of extensive animals testing, there is great interest in extrapolation of *in vitro* toxicity data (human and rodent systems) with PBPK models to calculate human equivalent doses for a given concentration *in vitro*. The corresponding NOAEC or AC_50_% (i.e., the concentration at 50% some specified maximal activity) could therefore be calculated using full-blown PBPK models or steady-state algorithms. The feasibility of using steady-state algorithms and *in vitro* screens to conduct *in vitro-in vivo* extrapolation for hazard ranking development has been reported [45, 46]. However, the adequacy of these approaches with regard to their assumption of 100% oral bioavailability and attainment of steady state during oral exposures needs to be further evaluated as a function of the information on chemical-specific dose metrics and MOA.

Although PBPK models and steady-state algorithms are designed to predict tissue and/or blood concentrations, further steps are needed to predict effects from internal doses. After estimation of internal dose from an external dose via PBPK modeling, biologically based dose-response (BBDR) models use internal dose estimates to predict response or toxicity through, for example, statistical correlations. Such BBDR models have been proposed as a computational tool for estimation of human risk. In general, there are a larger number of published PBPK models than BBDR models. The development of BBDR models is usually dependent on prior development of PBPK models; the added complexity and MOA information needed for BBDR model development may also hinder such model development. A BBDR model must also specify dosimetric and effect relationships across different species and exposure types in order to be considered successful, but such modeling does offer a unique opportunity to incorporate MOA information into one framework [47]. However, in addition to the uncertainty in both PBPK and BBDR models themselves, there is inherent uncertainty in the linkage of both components.

One of the major areas of BBDR development has been the quantification of cancer data through the use of two-stage clonal expansion models [48]. Although these BBDR models are biologically defensible, one of their inherent limitations is associated with the uncertainty of extrapolation from high doses (experimental data) to low doses (environmental exposure), as discussed by Crump et al. [49]. The technological problem becomes one of quantification of effect at low doses, where the noise in the data can overshadow the toxicological effect being measured. Hence, the advantage introduced by the addition of mechanistic steps into the model can be negated by both the inability to describe the dose response curve at low concentrations and to quantify the uncertainty introduced by the extrapolation process itself. These aspects of the process are not necessarily dependent on the biological mechanism used to describe the relationship.

BBDR model development can be used to investigate potential MOAs or the ability to identify data gaps. For a series of chemicals having a common MOA, a generalized BBDR model would be expected to explain relevant data for all chemicals. However, a recent application of BBDR modeling using a common liver MOA of action for several chemicals was successful in fitting the data for the individual chemicals but was not able to provide a generalized BBDR model common to all chemicals [50]. These authors indicated that, specifically for chloroform, the model may have oversimplified events leading to cancer. Although additional data may be helpful in establishing these details, the impact of uncertainty in the MOA should be addressed alongside BBDR model development [49].

Environmental exposures to contaminants do not occur in isolation but as mixtures. The health effects associated with mixture exposures are a result of not only the toxicity of each component, but also the interactions among the components. Even though the hazard characterization of individual specific chemicals has been the primary focus of numerous agencies (e.g., EPA and IARC), site-specific risk assessments are often for exposure to multiple chemicals. PBPK modeling approaches are continually being developed for mixtures. In this special issue, several papers addressed this subject (Sasso et al., Wang et al., and Becker et al.). Sasso et al. developed a PBPK model for a mixture of polychlorinated biphenyls. Complex total petroleum hydrocarbon (TPH) mixtures and their associated risks to human health were assessed by Wang et al. utilizing *in silico* or computational toxicological modeling approaches (i.e., comparative molecular field analysis and hierarchical clustering) in conjunction with established mixture risk assessment methods. Wang et al.'s *in silico* approach was compared to expert-driven judgment of fractionation of TPHs and their associated potential. The continued development of modeling approaches for complex mixtures is expected to both provide consistency in the appropriate grouping of chemicals in mixture analyses, and to predict the contribution of the individual chemicals to the overall toxicity.

On a broader scale, Becker et al. note that the evaluation of a large number of chemicals in commerce for potential human health risk has become a focus of attention in North America and Europe. Using translation from an external dose to a biomarker concentration framework, Becker et al. describe approaches for development of screening-level exposure guidance values. Specifically, applications of tools and concepts are discussed that include the threshold of toxicologic concern (TTC), biomonitoring equivalents (BEs), and generic toxicokinetic and physiologically based toxicokinetic models. 

## 5. Summary and Conclusions

Along with traditional uses of route-to-route and species-to-species extrapolations, PBPK models are being developed and tested for MOA applications and, based on biological sampling of metabolites and parent compounds in human tissue, reconstruction of exposure from model predictions. Increasingly sophisticated mathematical approaches and analyses are also being used to examine variability in pharmacokinetics. With less animal data available for model inputs, current efforts for screening and future efforts for new toxicology tests to replace animal testing are focusing on approaches based on either simplified models suitable for application to *in vitro* information or less-extensive animal data. In addition to *in vitro* to *in vivo* extrapolation, PBPK modeling applications continue to be developed as risk assessment tools for mixtures risk assessments, and developed for mechanism-based predictions that allow for linkage between internal dose-derived predictions (i.e. from PBPK models) and dose response (i.e. from BBDR models). In summary, as exemplified by the papers in this special issue, PBPK modeling provides tools for a wide spectrum of risk assessment applications that include (1) facilitation of the screening and prioritization of chemicals, (2) linkage of exposure to internal dose, (3) use in analysis of mechanistic information and prediction of risk to chemical mixtures, and (4) provision of computational techniques and tools to address uncertainty and variability questions related to identification and prediction of responses and pharmacokinetics in potentially sensitive subpopulations. 

## Abbreviations

1,4-D: 1,4-dioxane
ADME: Absorption, distribution, metabolism, and elimination
BEs: Biomonitoring equivalents
BPA: Bisphenol A
CYP: Cytochrome P450
DCM: Dichloromethane
EFSA: European Food Safety Authority
EPA: Environmental Protection Agency
HKAF: Human kinetic adjustment factor
IARC: International Agency for Research on Cancer
IPCS: International Program on Chemical Safety
IRIS: Integrated Risk Information System
MMT: Methylcyclopentadienyl manganese tricarbonyl
MTBE: Methyl tertiary butyl ether
MOA: Mode of action
NF*κ*B: Nuclear factor kappa-light-chain-enhancer of activated B cells
OECD: Organization for Economic Co-operation and Development
PBPK: Physiologically based pharmacokinetic
QSAR: Quantitative structure-activity relationship
RT-PCR: Real-time polymerase chain reaction
TDI: Tolerated daily intake
TPH: Total petroleum hydrocarbon
TTC: Threshold of toxicologic concern
VOCs: Volatile organic chemicals
WHO: World Health Organization.
